# Structure, Activation and Regulation of NLRP3 and AIM2 Inflammasomes

**DOI:** 10.3390/ijms22020872

**Published:** 2021-01-16

**Authors:** Meenakshi Sharma, Eva de Alba

**Affiliations:** Department of Bioengineering, School of Engineering, University of California, Merced, 5200 North Lake Road, Merced, CA 95343, USA; msharma32@ucmerced.edu

**Keywords:** inflammation, ASC (apoptosis-associated speck-like protein containing a CARD), NLRP3, AIM2, NMR, protein structure, protein assembly

## Abstract

The inflammasome is a three-component (sensor, adaptor, and effector) filamentous signaling platform that shields from multiple pathogenic infections by stimulating the proteolytical maturation of proinflammatory cytokines and pyroptotic cell death. The signaling process initiates with the detection of endogenous and/or external danger signals by specific sensors, followed by the nucleation and polymerization from sensor to downstream adaptor and then to the effector, caspase-1. Aberrant activation of inflammasomes promotes autoinflammatory diseases, cancer, neurodegeneration, and cardiometabolic disorders. Therefore, an equitable level of regulation is required to maintain the equilibrium between inflammasome activation and inhibition. Recent advancement in the structural and mechanistic understanding of inflammasome assembly potentiates the emergence of novel therapeutics against inflammasome-regulated diseases. In this review, we have comprehensively discussed the recent and updated insights into the structure of inflammasome components, their activation, interaction, mechanism of regulation, and finally, the formation of densely packed filamentous inflammasome complex that exists as micron-sized punctum in the cells and mediates the immune responses.

## 1. Introduction

Pathogen-associated molecular patterns (PAMPs) present in invading microbes and danger-associated molecular patterns (DAMPs) resulting from cellular insults are recognized by pathogen recognition receptors (PRRs). This recognition process results in the activation of a cytosolic supramolecular protein complex known as the inflammasome [1,2], which acts as a signaling platform and initiates an inflammatory response by triggering the production of proinflammatory cytokines (interleukin-1β (IL-1β) and interleukin-18 (IL-18)) [3]. Inflammasomes are divided into two categories, i.e., canonical inflammasomes, which activate caspase-1, and noncanonical inflammasomes that trigger activation of caspase-11 and caspase-4/5 in mouse and human, respectively [4,5,6]. The key components of canonical inflammasomes involve three classes of molecules, i.e., sensor, adaptor, and effector. These components tend to assemble via homotypic interactions between Death Domains (e.g., CARD-CARD (Caspase-activation and recruitment domain) and PYD-PYD (Pyrin domain)). In the presence of external stimuli or specific ligands, sensor proteins (ALRs (AIM2-like receptors) and NLRs (NOD-like receptors)) activate, oligomerize and nucleate the adaptor protein ASC (Apoptosis-associated speck-like protein containing a caspase-activation and recruitment domain (CARD)) through PYD-PYD interactions. ASC in turn recruits the effector protein procaspase-1 mediated by CARD-CARD homotypic recognition, thus serving as an activation platform of caspase-1.

Sensor proteins are divided into two classes: Absent in melanoma 2 (AIM2)-like receptors (ALRs) and NOD-like receptors (NLRs). AIM2 and IFI16 (Gamma-interferon-inducible protein) belong to the ALRs family and comprise an N-terminal PYD that interacts with ASC, and a C-terminal HIN (Hematopoietic, interferon-inducible, nuclear localization) domain for the recognition of double-stranded DNA (dsDNA) [7,8]. NLRs have tripartite domain organization and consist of: (1) N-terminal CARD or baculovirus inhibitor of apoptosis protein repeat (BIR) or PYD, which mediates homotypic protein–protein interactions for downstream signaling; (2) central nucleotide-binding domain (NBD) or NACHT domain, which elicits ATP-induced oligomerization; and (3) C-terminal manifold series of leucine-rich repeats (LRRs), which are responsible for ligand sensing and autoregulation [9,10,11,12]. The NBD domain belongs to the three subfamilies that include: NODs/NLRCs ((NLR family CARD domain-containing protein) NOD1–5), NLRPs/NALPs ((Nod-like receptor protein) NLRP1–14), and IPAF ((Ice protease activating factor) (NLRC4 and NAIP)). The human genome encodes for 23 NLRs; however, only a few NLR proteins such as NLRP1, NLRP3, NLRP6, NLRP7, NLRP9, NLRP12, and NLRC4 have been found to form inflammasomes and to activate caspase-1 [13,14,15,16,17,18]. These NLRs share structurally similar NACHT domains, but their activation is initiated by different stimuli [19,20]. A different type of inflammasome is formed by the sensor pyrin (also known as marenostrin or TRIM20), which contains a PYD domain, a B-box domain (zinc finger), a coiled-coil (CC) domain, and a B30.2/SPRY domain (absent in murine pyrin) [21]. Pyrin activates and assembles into the inflammasome complex in response to a bacterial infection that alters homeostasis and causes inactivation of RhoA GTPase [21]. Pyrin interacts with ASC through PYD-PYD homotypic interactions [21,22].

Upstream sensor proteins such as NLRP3 and AIM2 require the adaptor protein ASC for inflammasome assembly and activation, and thereby are known as ASC-dependent inflammasomes. Further homotypic interactions of ASC^CARD^ with caspase-1^CARD^ result in IL-1β and IL-18 release. These events facilitate the NF-κB (Nuclear factor kappa-light-chain-enhancer of activated B-cells), JNK (Jun N-terminal kinase), and p38 MAPK (Mitogen-activated protein kinase) signaling pathways that lead to high expression levels of cytokines and chemokines, and concomitant enlistment of immune cells to the site of pathogen invasion or tissue damage [23,24,25]. Other sensors with CARDs (e.g., NLRC4/NAIP and NLRP1) can directly activate caspase-1, and thus are representatives of ASC-independent inflammasomes [26,27]. However, reported studies on the function of the latter show that the presence of ASC enhances IL-1β secretion [28,29]. Details of activation signals, expression sites, and associated diseases of some canonical and non-canonical inflammasomes are summarized in Table 1.

Non-canonical inflammasomes are not inflammasome particles per se as described above. Instead, caspase oligomerization and autoactivation are mediated by direct binding of the CARD domain of caspase-11 with cell wall components of bacteria such as intracellular lipid A and lipopolysaccharide (LPS) [5,26]. This binding induces caspase oligomerization and activation, with the subsequent cleavage of gasdermin D (GSDMD) and cell death by pyroptosis [30].

Dysregulation of inflammasome activation is responsible for several autoinflammatory diseases that are associated with high levels of IL-1β and/or IL-18 secretion (Table 1). Currently, significant progress has been made in our understanding of the activation, assembly and regulation of inflammasomes. However, the study of the molecular basis responsible for inflammasome assembly and its dissolution is still an emerging field. Comprehensive structural and biophysical studies will help in the identification of the factors influencing inflammasome formation and disassembly, as well as in the discovery of therapeutic targets aimed at designing novel anti-inflammatory drugs. This review describes structural and biophysical insights into the NLRP3 and AIM2 inflammasomes.

## 2. NLRP3

### 2.1. General Information on NLRP3

NLRP3 is expressed in myeloid cells, muscle cells, neurons, and endocrine cells [98]. In the resting state, it exists as an autoinhibited form that becomes activated upon stimulation and assembles into a large, micrometer-size cytosolic complex. In macrophages, NLRP3 becomes functional in a two-step process that includes priming and activation. In the priming process, PRRs such as Toll-like receptors (TLRs), or NODs and cytokines such as TNF-α trigger the activation of the transcription factor NF-κB, thus affecting the expression of the inflammasome components NLRP3, caspase-1, and pro-IL-1β [99,100]. Subsequently, NLRP3 undergoes post-translation modifications (PTMs) (ubiquitylation, phosphorylation, and sumoylation) [101,102,103], which stabilize the protein in a signal-competent auto-suppressed inactive state, later to be transformed into an activated state upon stimulation. In the activation step, NLRP3 inflammasome assembles into a mature multiprotein-complex particle composed of NLRP3, ASC, and procaspase-1, which is capable of activating IL-1β and IL-18.

### 2.2. Role of the NLRP3 Inflammasome in COVID-19

COVID-19 (Coronavirus disease 2019), caused by the SARS-Cov-2 (Severe acute respiratory syndrome coronavirus 2) virus, was declared a pandemic by the WHO (World health organization), and as of 16 December 2020, approx. 73.5 million cases with 1.64 million deaths have been reported worldwide [104,105,106]. The pandemic has been associated with severe social and economic consequences. An upsurge of IL-1β, IL-18, and LDH (Lactate dehydrogenase) has been reported in sera of COVID-19 patients, which hints the involvement of the inflammasome network [107,108,109]. Similarly, a recent study conducted on COVID-19 patients reveals the activation of the NLRP3 inflammasome [110]. In this study, microscopic analysis in combination with luminescent assays show the formation of NLRP3 and ASC puncta, caspase-1 activation, and IL-1β secretion in PBMCs (Peripheral blood mononuclear cells) of COVID-19 patients during the disease and in postmortem lung tissues [110]. In addition, the level of Casp1p20 and IL-18 in COVID-19 patients has been shown as an important marker for determining disease severity [110].

### 2.3. Structural Details of NLRP3

**Figure 1:**
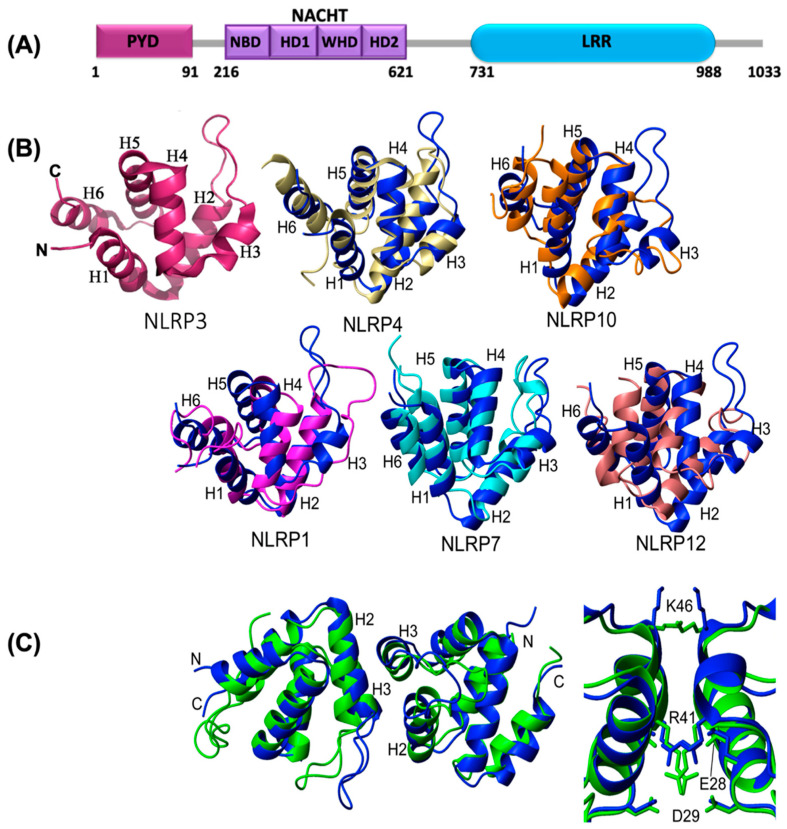
Structural details of NLRP3 and comparison with other pyrin domains (PYDs). (**A**) Schematic representation of NLRP3 domain organization; (**B**) 3D NMR solution-state structure of NLRP3^PYD^ (PDB ID: 2NAQ) [112]; superposition of 3D solution structures of different PYDs with NLRP3^PYD^ (blue, 2NAQ) which include NLRP1 (magenta, 1PN5); NLRP4, (ivory, 4EWI); NLRP7 (cyan, 2KM6); NLRP10 (orange, 2DO9); and NLRP12 (pink, 2L6A). Superimposed images are adapted from Figure 7A of [112]; (**C**) superposition of the crystallographic dimeric structure of NLRP3^PYD^ (green, 3QF2 [111]) onto the monomeric NMR structure (blue); the dimeric interface is shown in the right panel. These superposed images are adapted from Figure 7B of [112].

NLRP3 is composed of N-terminal PYD, central NACHT domain, and C-terminal LRR domain (Figure 1A). The structure of the PYD domain of human NLRP3 (NLRP3^PYD^) was determined by X-ray crystallography and solution-state NMR (Figure 1B,C) [111,112]. Both techniques reveal an overall similar architecture of the PYD domain with six helices (α1–α6) and five connecting loops, which is also analogous to the six-helix motif observed for the PYDs of NLRP1, NLRP4, NLRP7, NLRP10, and NLRP12 (Figure 1B) [111,112,113,114,115,116,117]. Among these, NLRP4 and NLRP10 show higher structural similarity with NLRP3 as compared to the other PYDs. However, pairwise structural alignments show that the orientation and length of the helices slightly differ in the NALP3^PYD^ structure. The six helices adopt a canonical anti-parallel helical-bundle fold tightly packed by a central hydrophobic core made up of helices α1 (L10, A11, Y13, L14), α2 (F25, L29), α4 (L54, A55, M58), α5 (I74, F75), and α6 (A87). In addition, NLRP3^PYD^ accommodates a second hydrophobic surface formed by F32, I39-P42, L57, and F61 residues [111], which stabilizes the shorter α3 helix by anchoring it to helix α2. Analysis of NLRP3^PYD^ electrostatic surface shows a charge distribution that might be involved in interactions with ASC^PYD^ or with other members of the Death Domain (DD) superfamily. In addition, structural homology and structure-based sequence alignment establish the presence of a conserved surface exposing a hydrophobic cluster (I39 and P40-P42 at α2–α3 loop and L57 and F61 at α4 helix) that could be responsible for inflammasome assembly and thus, caspase-1 activation [111]. The three-dimensional structures of NLRP3^PYD^ and ASC^PYD^, as well as studies on their interactions, reveal that both electrostatic and hydrophobic interactions play important roles. In addition, there are several surfaces in the PYDs of the two proteins available for the interaction, which potentially enhance oligomerization. Conserved residues in NLRP3, C8 (α1 helix), and C108 (loop connecting PYD and NACHT) form a unique disulfide bond that might be involved in ROS (Reactive oxygen species) signaling and NLRP3 inflammasome activation [111]. Based on X-ray crystallographic studies, it has been speculated that the formation of the disulfide bond relieves the autoinhibitory state of NLRP3 upon activation by ROS. NLRP3^PYD^ structures obtained from X-ray crystallography and solution-state NMR exhibit a high degree of resemblance (RMSD 1.66 Å); however, helices α2, α3, and α4 show slight differences between the crystal and solution structures (Figure 1C). Residue level calculation of B-factors obtained from X-ray crystallography suggests that NLRP3^PYD^ is highly compact in agreement with the NMR studies [112]. In particular, helices α2, α3, α4, and α5, and the two hydrophobic cores of the NLRP3^PYD^ are more rigid as reflected by the lower B-factor values, whereas the α2–α3 loop and C-terminus are more flexible.

**Figure 2:**
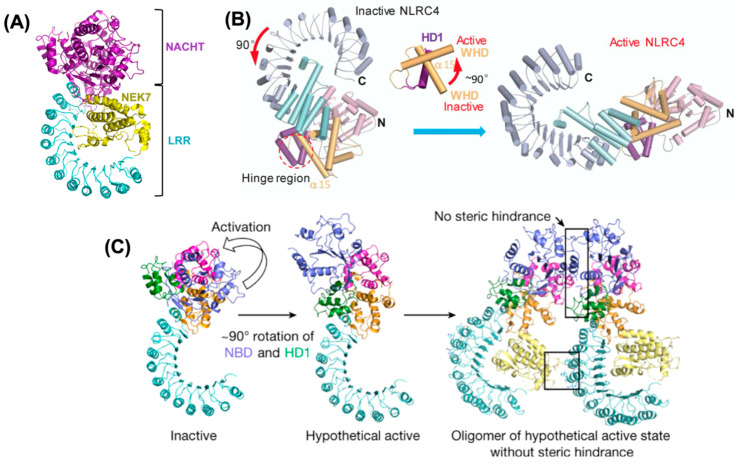
Activation of NLRC4 and NLRP3. (**A**) Cryo-EM structure of leucine-rich repeats (LRR) and NACHT domains of NLRP3 in association with NEK7 (PDB ID: 6NPY) [118]. The ribbon diagram is generated with PyMOL molecular graphics software; (**B**) structural organization and activation of NLRC4. This image is adapted from Figure 2C of [137]; (**C**) modelling of NLRP3 active conformation and NLRP3 dimer formation in association with NEK7. This image is adapted from Figure 5A,B of [118].

Recently, cryo-EM (electron microscopy) studies revealed that NLRP3 folds into a characteristic earring-shaped conformation (Figure 2A) also observed for NLRC4, NOD2, and NAIP5 (NLR family of apoptosis inhibitory protein) [118]. The structure has a curved LRR domain (12 repeats) and a compact NACHT domain comprised of NBD, HD1 (helical domain 1), WHD (winged helical domain), and a variable HD2 (helical domain 2) module (Figure 1A) [118].

### 2.4. Oligomerization of NLRP3^PYD^

The crystal structure of NLRP3^PYD^ reveals the presence of a symmetric dimer, whereas NMR, MALS (Multiangle light scattering), and SEC (Size-exclusion chromatography) show the coexistence of monomeric with higher-order oligomeric forms of NLRP3^PYD^ in solution. The existence of monomer or dimer depends on the concentrations of NLRP3^PYD^ and salt, as well as pH [111,112]. The crystal structure of the NLRP3^PYD^ (Figure 1C) dimer shows that residues E28 (α2), D29 (α2), and R41 (α3) in one protomer interact with the equivalent residues on the other protomer, which might result in repulsive interactions in the interface, thus suggesting that dimer structure is possibly an artifact from crystal packing [112]. The physiological relevance of the PYD dimer is still unclear. Some reports suggest that a PYD trimer, but not a dimer, can activate the inflammasome [119]; whereas others have found PYD dimer formation via a disulfide bond (e.g., NLRP12^PYD^) [120].

Sedimentation equilibrium and velocity results from analytical ultracentrifugation experiments and NMR studies indicate that NLRP3^PYD^ forms concentration-dependent oligomers [112]. At low protein concentration and acidic pH (0.2 mM, 303 K), NLRP3^PYD^ exists as a monomer that self-associates into higher-order oligomers at higher concentrations (0.6 mM, 303 K). The monomer–oligomer transition is drastically influenced by the presence of salt and temperature: Both the presence of salt and temperature increase favor oligomer formation even at low protein concentration (0.2 mM, 303 K, 100 mM NaCl). These results led to the conclusion that hydrophobic interactions play an important role in NLRP3^PYD^ self-association.

### 2.5. Activation and Regulation of NLRP3

#### 2.5.1. Post-Translational Modifications

Mutational studies uncovered that the LRR domain is not autoinhibitory because an LRR deleted variant demonstrates proinflammatory function similar to full-length (FL) NLRP3 in response to external stimuli [121]. These findings propose that the LRR domain is neither imperative for the assembly of active NLRP3 inflammasome nor crucial for the stabilization of the NLRP3 inactive state. It has been suggested that the importance of the LRR domain in NLRP3 activation could be related to: (1) Regulation by post-translational modifications due to the presence of ubiquitination and phosphorylation sites (K689 and Y861 in the human orthologue, and K687 and Y859 in the mouse orthologue) [121,122,123,124]; (2) the binding to NEK7, which is crucial for NLRP3 inflammasome activation [118]; and (3) the induction of T_H_2 cell (T helper type 2) responses [125]. T_H_2 cells facilitate adaptive immune responses against microorganisms and allergens by producing several interleukins (IL-4, IL-5, IL-9, IL-10, IL-13, and IL-25 (IL-17E)). The release of cytokines is further associated with antibody secretion, eosinophil/basophil activation, and various anti-inflammatory responses in order to provide phagocyte-independent protective responses. It is noteworthy that NLRP3 is also involved in the regulation of gene expression in T_H_2 cells in an inflammasome-independent manner. The transcription factor function of NLRP3 has been shown to be key for T_H_2 cell polarization. NLRP3 binds to DNA in T_H_2 cells at the Il4 promoter region and interacts with transcription factor IRF4 (Interferon regulatory factor) to regulate IL-4 synthesis. These results suggest that subcellular localization regulates NLRP3 functions by facilitating inflammasome assembly when expressed in the cytoplasm and promoting its transcriptional functions when localized in the nucleus.

Sequential phosphorylation–dephosphorylation events are also prerequisites for NLRP3 inflammasome activation. Several post-translational modification sites have been identified in human NLRP3 such as S5 (S3 in mouse), S198 (S194 in mouse), S295 (S291 in mouse), Y861 (Y859 in mouse), etc. [122,124,126,127]. Dephosphorylation by protein phosphatase 2 A (PP2A) at S5 located in the N-terminus PYD regulates the interaction of NLRP3 and ASC [122]. It was found that a phosphomimetic mutant of S5 that creates a negative charge completely attenuates NLRP3 activation by disturbing the interaction between NLRP3 and ASC [122]. Similar results were obtained from the phosphorylation of S295 by protein kinase A (PKA) and protein kinase D (PKD) [127,128]. In addition, dephosphorylation of Y861 by PTPN22 allows NLRP3 activation and subsequent IL-1β secretion [124]. The absence of PTPN22 in cells results in increased NLRP3 phosphorylation, which abolishes inflammasome assembly and reduces IL-1β secretion [124]. Another kinase, Jun N-terminal kinase1 (JNK1), has been shown to phosphorylate S198, providing a critical priming signal for NLRP3 self-association [126]. The phosphorylation/dephosphorylation interplay has been associated with the cryopyrin-associated periodic syndrome (CAPS), as it has been shown to be coupled to impaired phosphorylation at the S194 site in mouse [126]. Hence, the inhibition of NLRP3 phosphorylation/dephosphorylation processes is a potential pharmaceutical target for the treatment of NLRP3-associated diseases [126].

Ubiquitination and deubiquitination events are crucial for NLRP3 inflammasome regulation and involve a series of enzymatic steps mediated by ubiquitin-activating enzyme (E1)-ubiquitin-conjugating enzyme (E2)-ubiquitin ligase (E3), and deubiquitinating enzyme (DUBs), respectively [103,123]. The SCF (Skp-Cullin-F box) family member, FBXL2 (F-box L), is an anti-inflammatory molecule that binds at the W73 site and targets K689 for ubiquitin ligation and proteasomal degradation of NLRP3. In response to LPS exposure, an elevated level of FBXO3 (F-box O3), an E3 ligase, inhibits FBXL2 that in turn upregulates the NLRP3 expression level in cells and increases the release of proinflammatory cytokines. A small molecule, BC-1215, acts as an inhibitor for FBXO3 and reduces the secretion of matured IL-1β and IL-18 [123]. It has also been reported that neurotransmitter dopamine (DA) activates the ubiquitin E3 ligase, MARCH7 (membrane-associated ring finger (C3HC4) 7), that inhibits NLRP3 inflammasome activation via K48-linked polyubiquitination [129]. Similarly, another E3 ubiquitin ligase, Pellino2, assists in K63-linked ubiquitination and induces NLRP3 activation [130].

BRCC3 (BRCA1/BRCA2- containing complex, subunit 3), a murine deubiquitinating enzyme (human orthologue is BRCC36), is also involved in regulating NLRP3 activity. BRCC3, in combination with the protein ABRO1 (Abraxas brother 1), forms the cytosolic BRISC complex and deubiquitinates the LRR domain of NLRP3 by specifically cleaving the K63-linked polyubiquitin chain [103]. The ubiquitin isopeptidase inhibitor, G5 (NSC 144303), acts as an inhibitor for DUBs and inhibits deubiquitination and subsequent NLRP3 activation [103]. The protein NleA, secreted by enteropathogenic and enterohemorrhagic *E. coli*, inhibits deubiquitination of NLRP3 to limit inflammasome activation by hijacking the ubiquitin machinery [131]. A20, a ubiquitin modifying enzyme, acts as a negative regulator of NLRP3 [132,133]. Similarly, TRIM31 (tripartite motif 31) induces K48-linked ubiquitination and subsequent proteasomal degradation of NLRP3 [134]. Another E3 ubiquitin ligase, Shigella IpaH7.8, also activates NLRP3-inflammasome by targeting glomulin (GLMN), which is a member of Cullin ring ligase inhibitor [135].

#### 2.5.2. NEK7 Mediated Activation of NLRP3

NIMA (Never In Mitosis gene A)-related kinase 7 or mitotic Ser/Thr kinase NEK7 regulates NLRP3 activation [118]. Cryo-EM structural analysis of the complex formed between NLRP3 without the PYD, the NEK7 C-lobe, and ADP bound to the NBD of NLRP3, reveals that the C-terminal lobe of NEK7 interacts with the NBD, HD2, and LRR regions of NLRP3 (Figure 2A). In addition, the dissociation constant of the complex between NLRP3 and NEK7 was determined to be, K_d_ 78.9 ± 38.5 nM. The binding between NLRP3 and NEK7 involves two interfaces: The first and second half of the NEK7 C-lobe interact with the LRR and NACHT domains (NBD and HD2), respectively. Residues Q129, R131, and R136 of NEK7 interact with the LRR domain, whereas residues D261, E265, and E266 interact with HD2, and D290, K293, and R294 interact with the NBD. Mutagenesis studies showed that both interfaces are required for NLRPE3-NEK7 complex formation. The substitution of G755 in LRR for the amino acids A or R leads to enhanced interaction between LRR and NEK7 [136]. In contrast, phosphorylation of Y859, located in the LRR domain, causes steric hindrance and charge repulsion, thus aborting the interaction with NEK7 [124].

A computational model of the oligomeric assembly of the NLRP3-NEK7 complex was generated to understand the NLRP3 activation mechanism using the structure of a full-length NLRC4 oligomer as a template [118]. It has been reported that the inactive form of NLRC4 undergoes a 90° rotation of the NBD-HD1 module with respect to the WHD-HD2-LRR module, which generates an active NLRC4 conformation (Figure 2B) [137,138]. The conformation of the NBD-HD1-WHD module of NLRP3 is similar to that of the inactive form of NLRC4; therefore, the active oligomeric structure of NLRP3 has been modeled from its inactive form by using the NLRC4 activation mechanism (Figure 2C) [118].

#### 2.5.3. Role of Caspase-8 in Inflammasome Activation

Caspase-8 plays an important role in the regulation of inflammatory responses by direct cleavage of pro-inflammatory cytokines into their mature forms, and by activating the NLRP3 inflammasome [139,140]. Caspase-8 consists of a death effector domain (DED) at the N-terminus, and p18 and p10 catalytic subunits at the C-terminus. Cell studies showed that in the absence of caspase-1, pro-IL-1β processing is mediated by caspase-8 in response to LPS [141], and to a wide range of stimuli, such as activation of TLR4 in bone marrow-derived dendritic cells (BMDCs) [142] and Fas death receptor. Ligation to the bacterial and fungal C-type lectin receptor, dectin-1, triggers caspase-8 activation via CARD9-Bcl-10-MALT1 complex. Other stimuli include endoplasmic reticulum stress, chemotherapeutic agents, inhibition of c-FLIP (FLICE-like inhibitory protein), and histone deacetylases (HDAC) [143,144,145,146,147,148]. Activated caspase-8 cleaves pro-IL-1β at the same site (D117) utilized by caspase-1 [141,149].

Caspase-8 acts as positive and negative regulator of the NLRP3 inflammasome depending on specific cell types. In macrophages, caspase-8 together with FADD (Fas-associated protein with death domain) drives the priming and activation of the canonical and non-canonical inflammasomes [150]. It has been shown that in the absence of IAPs (inhibitor of apoptosis proteins), LPS or TNF-primed murine macrophages and dendritic cells show TLR-TRIF (TIR-domain-containing adapter-inducing interferon-β)-RIPK1 (Receptor-interacting protein kinase-1)-RIPK3-caspase-8 mediated activation of the NLRP3 inflammasome [142,151,152]. Similarly, human BlaER1 monocytes show TLR3 activation mediated by the TRIF-RIPK1-FADD-caspase-8 pathway [153]. In contrast, caspase-8 deficient murine BMDCs and macrophages, and human BlaER1 monocytes show RIPK1-RIPK3 dependent necroptotic activation of the NLRP3 inflammasome upon TLR4 ligation [152,154,155]. Ablation of IAPs and caspase-8 in TLR-primed macrophages and BMDCs leads to NLRP3 activation by RIPK3-MLK (Mixed lineage kinase domain-like pseudokinase), which suggests a negative role of caspase-8 in inflammasome activation [152,156]. In dendritic cells, it inhibits RIPK1-RIPK3-MLKL-mediated NLRP3 inflammasome activation [152,154]. Some studies indicate that caspase-8 is involved in pore formation in the plasma membrane by activating GSDMA or pannexin-1 (channel-forming protein) that in turn facilitates NLRP3 inflammasome activation via K^+^ efflux [157,158]. It has also been reported that caspase-8 is an integral part of the inflammasome particle, comprising MALT1, caspase-8, and ASC, and directly processes pro-IL-1β without caspase-1 involvement [159]. Caspase-8 interacts with ASC^PYD^ via DED, which induces caspase-8 polymerization and subsequent activation of ASC [160].

## 3. AIM2

**Figure 3:**
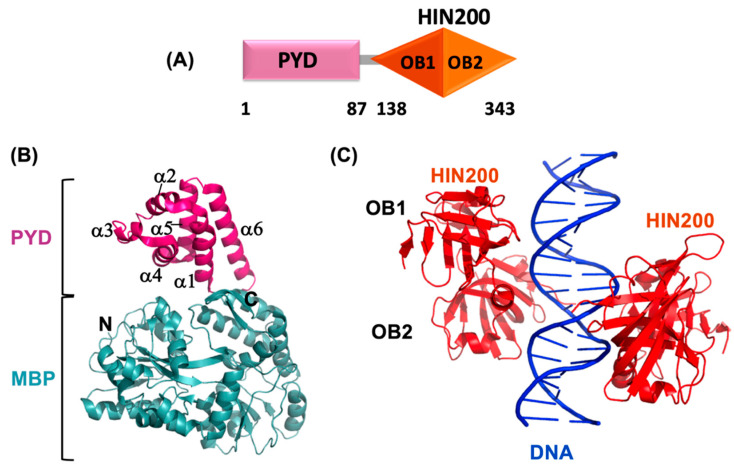
Structural details of AIM2. (**A**) Schematic representation of AIM2 domain organization; (**B**) crystal structure of AIM2^PYD^ with Maltose-binding protein (MBP) fusion tag (PDB ID: 3VD8) [161]; (**C**) crystal structure of AIM2^HIN^ in complex with dsDNA (PDB ID: 3RN2) [162]. The ribbon diagrams are generated with PyMOL molecular graphics software.

AIM2 is a cytosolic dsDNA sensor that is responsible for downstream signaling to the adaptor protein ASC in response to the presence of bacterial and viral DNA. AIM2 belongs to the PYHIN family (pyrin + HIN) and consists of an N-terminal PYD (1–87) and a C-terminal HIN domain (138–343) connected through a long linker [161,162] (Figure 3A). AIM2 interacts with ASC via PYD-PYD homotypic interaction and the HIN domain binds to dsDNA in a sequence-independent manner. In addition, AIM2 also heterodimerizes with other members of the PYHIN family such as p202, IFI16, and MNDA (Myeloid cell nuclear differentiation antigen) [69,163,164,165]. AIM2 interaction with ASC further activates procaspase-1 leading to pyroptosis, whereas the interaction with p202 inhibits AIM2-mediated inflammatory responses [68,166,167,168].

### 3.1. AIM2^PYD^ Domain Structure

The crystal structure of AIM2^PYD^ reveals the six-helix bundle conformation characteristic of the DD superfamily [161] (Figure 3B). It shares structural homology with PYDs of NLRP3 and ASC with RMSD values of 1.8 Å and 1.6 Å, respectively [161]. However, AIM2^PYD^ exhibits a short and highly dynamic α3 helix and long α6 helix with the most variable sequence among the known PYDs. Largely buried and highly conserved K26 residue located at α2 helix buttresses α3 helix via hydrogen bonding with L40 and A43 of the interconnecting loop, thereby stabilizing the α3 helix. The overall surface of AIM2^PYD^ is populated by charged residues, thus resulting in distinct electrostatic charge distribution. These amino acids include acidic residues such as E7 and D15 in α1 helix; D19, E20, E21, and D23 in α2 helix; and basic residues such as K64, R67, and K71 in α5 helix; and K79, R80, K85, K87, K90, K93, and K97 in α6 helix. Among these, K64 and K85 are conserved in the PYHIN family, and E20 and E21 are specific to AIM2. Residues such as F27 and F28 create a solvent-exposed hydrophobic patch that shares similarities with the DED surface involved in homotypic interactions, suggesting that they may contribute to AIM2^PYD^ self-association and AIM2-specific functions [161].

AIM2^PYD^ tends to form large, insoluble oligomers in solution, and thus poses significant challenges for biophysical studies. To overcome oligomerization, AIM2^PYD^ has been fused to an MBP (Maltose-binding protein) tag and a specific mutant, F27G, has been created to shift the monomer–oligomer equilibrium to the monomeric form [169]. The crystal structures of mouse AIM2^PYD^ (mAIM2^PYD^), wild-type (WT) human AIM2^PYD^ (hAIM2^PYD^), and hAIM2^PYD^ F27G mutant very similar, as expected for an amino acid sequence identity of 56%. However, the conformational arrangement of helices α2 and α3 shows significant differences [169,170]. Helix α3 of mAIM2^PYD^ is positioned adjacent to the N-terminus of helix α2, similarly to the reported conformation of these helices for hNLRP10^PYD^ [169,170]. In contrast, helix α3 is positioned adjacent to the C-terminus of helix α2 in hAIM2^PYD^ F27G mutant and shows intermediate orientation in the structure of WT hAIM2^PYD^. Differences in chain flexibility have also been observed; for example, the α2-α3 helical region is relatively ordered, with an average B-factor of 18.7 Å^2^ in hAIM2^PYD^ F27G mutant and of ~120 Å^2^ in wild type AIM2^PYD^ [169].

### 3.2. AIM2^PYD^ Self-Association

Isolated AIM2^PYD^ can self-associate and form filaments similar to ASC^PYD^. Homology modeling of the AIM2^PYD^ filament using the cryo-EM structure of the ASC^PYD^ filament as a template in combination with negative stating (ns)-EM data revealed that the AIM2^PYD^ filament shows a three-fold symmetry arrangement of the PYD protomer structures [167,171]. EM analysis proposes that the AIM2^PYD^ filament serves as a structural template for ASC polymerization [167]. It has been reported that mAIM2^PYD^ maintains monomeric conformation at low pH (4.0) and low salt concentration (< 100 mM) due to repulsive electrostatic forces between positively charged molecules and by interfering with hydrophobic interactions, whereas high salt concentration promotes oligomerization through hydrophobic interaction of hydrophobic patches on the protein surface [170]. These results confirm that both electrostatic and hydrophobic interactions are necessary for AIM2^PYD^ polymerization, which was also observed for the interaction between the PYDs of ASC and NLRP3 and for ASC^PYD^ self-association [121,169,171]. Isolated AIM2^HIN^ cannot form ordered macrostructures, thus pointing to a predominant role of Death Domains such as PYD in the formation of ordered polymers. In fact, AIM2^PYD^ self-association has been shown to suffice for inducing the assembly and activation of the inflammasome [169].

Cryo-EM structural studies of GFP-AIM2^PYD^ filament indicate that this truncated construct forms ~200 nm to ~1 μm long filaments with outer and inner diameters of ~90 Å and ~20 Å, respectively. In this filament structure, the GFP tag protrudes from a filament core formed by AIM2^PYD^ [172]. Modeling studies propose that type I, II, and III interactions characteristic of Death Domains (PYDs and CARDs) play important roles in the helical organization of the AIM2^PYD^ filament. In the type I interaction, residues of helices α1 and α4 (S3, K6, L10, L11, D31, and I46) located on the first protomer interact with residues on helices α2 and α3 (R24, F27, F28, and D31) located on the fourth protomer. In the type II interaction, residues of helix α4 (Q54 and N55) located on the first protomer interact with residues of helices α5 and α6 (N73, Y74, and L76) located on the sixth protomer. In type III interaction, residues of helix α3 (G38 and K39) of the first subunit interact with residues in helices α1 and α6 (D15, N16 and I17) of the third protomer. The type I interaction is mediated by hydrophobic contacts and dominates filament assembly. Thus, mutations of residues such as F27G/F27L (type Ib surface) and L10A/L11A (type Ia surface) inhibit AIM2^PYD^ self-association and promote the monomeric form. Other structural studies have used the MBP fused to AIM2^PYD^ to impede oligomerization. It has been found that residues L10 and L11 are located near the MBP in this construct, which agrees with the finding that these residues are involved in oligomerization via the type I interface [172].

### 3.3. AIM ^HIN^ Domain Structure

The HIN domain of AIM2, with ~200 amino acids, comprises two tandem OB (oligonucleotide/oligosaccharide binding) folds connected through a long linker (Figure 3C). Canonical OB folds contain five β-strands that fold into two sheets [162]. The proximal OB1 fold consists of β1–β5, among them β1, β4, and β5 split into two short strands (β and β’). Similarly, the distal OB2 fold (β1–β5) shows the splitting of β5 into two shorter strands. The linker connecting OB1 and OB2 is ~30 residues long and is folded into two alpha-helices. The two OB folds firmly interact with one another through conserved hydrophobic interactions. The HIN domains of AIM2, IFI16, and p202 are highly conserved and show an identical topological arrangement of OB folds [162,173].

### 3.4. AIM2 HIN:dsDNA Interaction

The crystal structure of the AIM2^HIN^ domain in complex with dsDNA derived from the *Vaccinia* virus was determined using X-ray crystallography [162] (Figure 3C). This structure reveals that the highly positively charged surface of HIN interacts with the sugar-phosphate backbone of dsDNA mainly via electrostatic interactions. The N-terminus of the HIN domain is positioned far away from the DNA-binding surface, possibly facilitating the interaction between the N-terminal PYD of AIM2 with the adaptor protein ASC. The binding of the HIN domain to both the major and minor grooves of the DNA could explain AIM2-induced activation of the innate immune system in the presence of dsDNA, but not ssDNA [66,68,162]. Both OB folds and the connecting linker participate in DNA binding. Specifically, residues K160 (β1), K162 (β1), and K163 (β1–β1’ loop) of the OB1 fold, as well as residues L267 (β1), N287 (β2), K309 (β4), R311 (β4), K335 (β5), and I337 (β5), of the OB2 fold and linker residues such as R244 (α2), G247 (α2), and E248, T249, and K251 located at α2–α3 loop, participate in the binding between AIM2^HIN^ domain and dsDNA mainly via hydrogen bonding, van der Waals interactions, and salt bridges. The crystal structure also shows the formation of bidentate hydrogen bonds between residue R311 and phosphate groups in the DNA backbone [162].

It has been reported that ~80 bp of dsDNA is the minimum size required for the induction of IL-1β by AIM2 activation [162]. Each HIN domain occupies four DNA base pairs, hence ~20 AIM2^HIN^ domains wrap around the 80 bp of dsDNA with an observed axis tilt of 35°. Multiple sequence alignment suggests that most residues interacting with dsDNA are also conserved in IFI16^HIN^ and mouse AIM2^HIN^ domains. Site-directed mutagenesis studies indicate that mutations involving residues located on the interacting regions, such as the OB1-linker, the OB1-linker-OB2, and residue F165, lead to a diminished binding affinity of AIM2^HIN^ to dsDNA. These results on AIM2^HIN^ were corroborated by similar mutagenesis experiments conducted on full-length AIM2 (AIM2^FL^), which resulted in an impaired association of AIM2 with DNA and reduced IL-1β secretion [162]. Altogether, these studies suggest that an intact receptor binding surface is required for the association to dsDNA and the subsequent immune responses.

### 3.5. AIM2 PYD:HIN Interaction

Xiao et al. originally proposed that in the absence of a ligand, intramolecular interactions between the PYD and HIN domains in AIM2 retain the sensor in an autoinhibited state that prevents PYD-mediated oligomerization and suppresses HIN:DNA binding [162].

Docking analyses of crystal structures suggest that the negatively charged helix α2 of the PYD locates at the interface of the PYD:HIN interaction. In addition, ITC (Isothermal titration calorimetry) studies reveal that AIM2^PYD^ interacts with AIM2^HIN^ with a dissociation constant (K_d_) of 23.5 µM. Furthermore, it has been shown that mutations of acidic residues located in helix α2 abolish the binding of the PYD and HIN domains [161]. These results confirm that the PYD-HIN interface is dominated by electrostatic interactions between negatively and positively charged residues in the PYD and HIN domains, respectively. The negatively charged surface of AIM2^PYD^ that participates in the interaction with AIM2^HIN^ is also involved in the binding to ASC^PYD^, hence ensuring downstream signaling to the adaptor ASC only after AIM2 is activated by dsDNA.

### 3.6. Importance of AIM2^PYD^ in dsDNA Interaction and Oligomerization

The hypothesis of the autoinhibitory model was challenged by Sohn and colleagues [171]. Based on their studies, they proposed that AIM2^PYD^ does not participate in AIM2 autoinhibition, instead, it actively helps in DNA binding and concomitant self-association. We mentioned above that the fusion of MBP to the N-terminus of AIM2^FL^ interferes with PYD oligomerization. To interrogate whether the PYD has a role in dsDNA binding, fluorescence anisotropy experiments were conducted to compare the affinity of fluorescein amidite (FAM)-labeled 72-bp dsDNA with MBP-AIM2^FL^, MBP-AIM2^HIN^, untagged AIM2^FL^, and untagged AIM2^HIN^. The results show that MBP-AIM2^FL^ binds 2-fold tighter to dsDNA than MBP-AIM2^HIN^, whereas untagged AIM2^FL^ binds at least 20-fold more tightly than MBP-tagged AIM2 variants in presence of 160 mM KCl. Another important finding from the Sohn group is that the isolated HIN domain can oligomerize upon dsDNA binding and thus assists in filament formation. Salt concentration-dependent binding reveals that AIM2^HIN^ oligomerizes on dsDNA in presence of 160 mM KCl, but fails to bind at 400 mM KCl. On the other hand, AIM2^FL^ binds to dsDNA even at this high salt concentration, which hints the involvement of PYD in dsDNA binding. Furthermore, mutations of residues L10, L11, and F27 involved in AIM2^PYD^ self-association and non-conservative mutations of AIM2^PYD^ acidic residues D19, E20, E21, and D23 impede the binding of AIM2^FL^ to dsDNA at 400 mM KCl, further supporting that the oligomerization of AIM2^PYD^ plays an important role in dsDNA binding. Although the effects of these mutations in the 3D-fold of the PYD were not tested, it would be expected that these residues facilitate the transformation of AIM2 from the autoinhibited conformation to the activated form that can bind DNA even at high salt concentration. Therefore, the results obtained with these mutants contradict the inhibitory role of the AIM2^PYD^ in DNA binding.

AIM2^FL^ needs to bind to a larger dsDNA size (~12 bp) as compare to the HIN domain alone (~8 bp), confirming the relevance of oligomerization to potentiate dsDNA binding. Furthermore, binding of AIM2^FL^ to dsDNA increases 1000-fold in the presence of 10-times longer DNA, indicating cooperativity between dsDNA size and AIM2 binding affinity. For the formation of AIM2^FL^-dsDNA complex, ~70 bp dsDNA and six molecules of AIM2^FL^ are needed to cross the binding threshold (lag phase), and ~250–300 bp dsDNA and ~24 AIM2^FL^ molecules are required for establishing an optimal oligomeric complex, as determined by fluorescence anisotropy competition binding assays using FAM-dsVACV72 (1.5 nM) and AIM2^FL^ (70 nM) against various fragments of dsDNA at 400 mM KCl. The data were fit to competition binding equation; $1/[1+\left(\left[DNA_{competitor}\right]/IC_{50}{)}^{Hill\;constant}\right]$ [171]. This observation is further confirmed by monitoring the increase of IL-1β secretion with increasing dsDNA size. Overall, these results propose that dsDNA size acts as a ‘molecular ruler’ to regulate AIM2 inflammasome assembly in a switch-like mechanism of PYD oligomerization and dsDNA binding.

In the presence of dsDNA excess, AIM2^FL^ shows saturating or increased size-dependent FRET (Fluorescence resonance energy transfer) signals, but AIM2^HIN^ displays decreased FRET signals, indicating that AIM2^PYD^ is key for dsDNA binding and promotes oligomerization in presence of dsDNA excess [171]. Interestingly, ns-EM images illustrate that AIM2^FL^ is able to self-oligomerize in the absence of dsDNA forming “Brussels sprout-like” filaments at high concentration (≥500 nM) [171]. Oligomerized AIM2^PYD^ forms the filament core (~9 nm) and the HIN domains are observed at the periphery of this core, like Brussel sprouts. In contrast, filaments formed by AIM2^FL^ bound to dsDNA are ~25 nm wide. The participation of the PYD in filament assembly is critical, as isolated AIM2^HIN^ and MBP-AIM2^FL^ do not show any ordered filament formation in absence of dsDNA, and isolated AIM2^HIN^ displays random ‘beads on a string’-like cluster upon dsDNA addition. Moreover, mutagenesis studies show that both PYD and HIN domains are required for the oligomerization of AIM2^FL^ in the presence or absence of dsDNA. FRET results reveal that the length of dsDNA regulates the assembly kinetics and lifetime of the dsDNA-AIM2 complex [174]. Modeling analysis based on cryo-EM and ns-EM observations propose that in the dsDNA-AIM2^HIN^ filament complex of ~7.5 nm diameter, AIM2^HIN^ is wrapped around the dsDNA core and each HIN molecule interacts with six adjacent HIN molecules [172]. Such an arrangement of AIM2^HIN^ around the DNA core and long linker between both domains brings AIM2^PYDs^ into close proximity where they form short helical filaments proposed to run parallel to the DNA and to act as a platform for ASC^PYD^ filament nucleation.

Altogether, these results propose that in the absence of cytosolic dsDNA, AIM2 is expressed at a very low basal concentration level, and is therefore unable to oligomerize and induce downstream signaling via ASC. Pathogenic attack facilitates rapid oligomerization due to invasion of dsDNA in the cytosol, which hikes AIM2 local concentration [171]. The size of dsDNA acts as a molecular ruler and governs the AIM2 assembly.

### 3.7. Regulation of AIM2

#### 3.7.1. Negative Regulators of AIM2 Inflammasome Activation

Regulation of inflammasome assembly is imperative for maintaining cellular homeostasis. The mouse protein p202 has been reported to sequester cytoplasmic dsDNA and inhibit AIM2 activation [69]. p202 consists of two HIN domains and lacks the PYD, rendering it unable to recruit ASC. The binding of p202 to DNA and AIM2 is proposed to attain a balance between pathological DNA-induced inflammation and physiological host defense. The crystal structure of mouse p202-dsDNA complex reveals that the p202^HIN1^ domain binds to DNA, whereas p202^HIN2^ interacts with AIM2 [173]. Full-length p202 (p202^FL^) forms a tetramer in cells as well as in vitro purified protein solutions. p202^HIN2^ first dimerizes in a parallel fashion using both OB folds (OB1-OB2 to OB1-OB2) and the formed dimers assemble into tetramers in a tail-to-tail orientation of the OB2 folds (OB2 to OB2) [166]. Although p202^HIN2^ lacks DNA binding capability, tetramer formation serves as a platform for p202^HIN1^ attachment to dsDNA, increasing the overall DNA binding affinity of p202^FL^ as compared to AIM2. p202^HIN1^ shares structural similarity with mAIM2^HIN^ and IFI16^HIN2^, but shows different charge distribution and opposite orientation of the dsDNA binding surface. Such a difference in surface electrostatic potential is responsible for the antagonist activity of p202 [166].

Unlike AIM2, the linker connecting the two OB folds of p202 does not participate in DNA binding. Positively charged residues located at the N-terminus and loop between β1–β2 of the OB1 fold engage with the DNA minor groove [166]. In the OB2 fold, residues located in the loop connecting β1–β2 and the loop between β4–β5 interact with the dsDNA major groove. Structure-based mutagenesis studies propose that among these, OB1 N-terminal residues K48, and K53, and OB2 residue R224 are crucial for HIN:DNA interactions. Most of these residues interact with the backbone of DNA; however, K53 side chain was found to make two hydrogen bonds with DNA bases.

p202^HIN2^ interacts with AIM2 through a short sequence motif (MFHATVAT) conserved in both proteins and buried in the core of the HIN domains [175,176]. The protein region, MFHATVAT, is required for p202 dimerization and subsequent interaction with AIM2 [175]. It has been reported that p202^HIN2^ does not block the DNA binding surface of AIM2; therefore, DNA binding affinity of AIM2 remains unaffected in the presence of p202^HIN2^ [166]. Computational docking studies showed that the binding of AIM2^HIN^ domains with both ends of the p202^HIN2^ tetramer creates a spatial separation between AIM2^PYDs^, thus preventing ASC oligomerization [166]. Consequently, the knockdown of p202 increases the level of ASC and cross-linked ASC oligomers [166]. In this line, modeling studies propose that two adjacent mouse AIM2 (mAIM2) molecules bound to DNA are separated by less than 10 Å, thus generating AIM2 molecular crowding and favoring the interaction with ASC^PYD^ and the subsequent activation of inflammasome assembly [173]. In contrast, p202 spans a larger dsDNA fragment and binds with higher affinity compared to AIM2. Therefore, when both p202 and AIM2 are present in equal amounts, the former competes with the latter for dsDNA binding and covers a larger surface area of dsDNA [173].

#### 3.7.2. IFI16-β Mediated Regulation of AIM2 Inflammasome Activation

A novel human isoform of IFI16 designated as IFI16-β has been shown to selectively inhibit the formation and activation of AIM2 inflammasome assembly [177]. IFI16-β is ubiquitously expressed in various human cells and shows upsurge expression in leukocytes in case of viral infection. IFI16-β co-localizes with AIM2 in the cytoplasm and by sequestering cytoplasmic dsDNA, impedes its detection by AIM2. Analogously to p202, IFI16-β contains two HIN domains (HIN A and HIN B) and disrupts AIM2-ASC inflammasome activation by interacting with AIM2, competing with dsDNA binding as well as inhibiting AIM2 oligomerization [166,173,177]. Competition binding experiments suggest that IFI16-β binds with higher affinity to dsDNA than AIM2 because the IFI16-β-DNA complex shows a more prominent band in biotin-dsDNA pull-down assays as compared to AIM2 [177]. Altogether, dsDNA binding studies of p202 and IFI16-β indicate that proteins expressing two HIN domains bind to dsDNA more robustly than single HIN domain-containing proteins like AIM2 [166,173,177].

#### 3.7.3. Post-Translational Modifications of AIM2

Very limited information is available on AIM2 post-translation modifications. However, it has been reported that TRIM11 (tripartite motif 11) acts as a negative regulator of the AIM2 inflammasome. TRIM11 binds AIM2 and undergoes poly-ubiquitination at K458, leading to the recruitment of autophagy cargo receptor p62, thus mediating the subsequent degradation of AIM2 [178,179]. In addition, studies conducted on mouse models of stroke and cultured primary microglia show elevated expression of HDAC3 (Histone deacetylases 3) linked to the regulation of the inflammatory process by activating the AIM2 inflammasome. RGFP966, a HDAC3 inhibitor, downregulates the AIM2 inflammasome by enhancing acetylation and inhibiting phosphorylation (at Y701 and S727) of STAT1 (Signal transducer and activator of transcription) in order to protect against ischemic brain injury [180].

## 4. ASC

**Figure 4:**
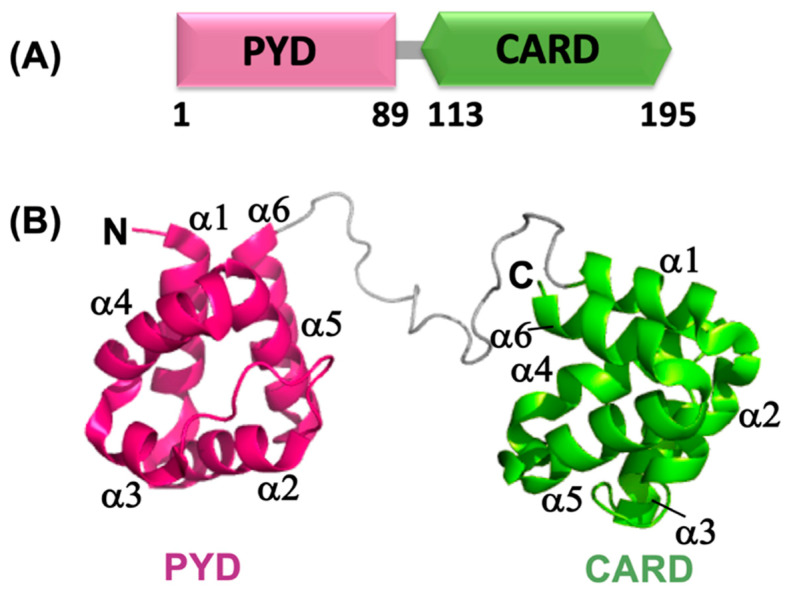
Structural details of ASC. (**A**) Schematic representation of ASC domain organization; (**B**) 3D NMR solution-state structure of full-length ASC (PDB ID: 2KN6) [183]. The ribbon diagram is generated with PyMOL molecular graphics software.

ASC (PYCARD; PYD and CARD domain-containing or TMS; Target of Methylation-induced Silencing-1) is a ~24 kD bifunctional cytosolic adaptor protein that consists of an N-terminal PYD (1–89) and a C-terminal CARD (113–195) connected by a 23 residue-long linker (90–112) [181,182,183] (Figure 4A). ASC expresses in the nucleus of epithelial and immune cells, and in response to inflammatory stimuli, is redistributed to the cytoplasm where it assembles into a compact micrometer-size perinuclear structure referred to as ASC speck or ASC foci [181,182,184]. The ASC speck colocalizes with the sensor and the procaspase-1 by homophilic interactions mediated by the PYD and CARD domains, thus forming the inflammasome, which serves as the platform for caspase activation and pyroptotic cell death [168,185,186,187]. In addition to ASC^FL^, three other isoforms also exist: ASC-b, which also bears an N-terminal PYD and C-terminal CARD as with ASC^FL^, although connected by a short 3 amino acid-long linker; ASC-c, retaining the CARD, but only a partial PYD; and ASC-d, a 105-amino acid long polypeptide that only conserves residues 1–35 of the original ASC^FL^ sequence [188]. These isoforms respond differently to inflammatory stimuli, exhibit irregularly shaped perinuclear aggregates and differential cellular expressions.

ASC^PYD^ interacts with the PYDs of NLRP3 and AIM2, whereas ASC^CARD^ interacts with the CARDs of procaspase-1 and NLRC4 via homotypic interactions [20,189]. The 3D NMR-solution structure of ASC reveals that the PYD and CARD domains form rigid structures with RMDS of 0.78 ± 0.07 and 0.79 ± 0.08 Å, respectively. The two Death Domains do not interact with one another based on Nuclear Overhauser data (NOE) and are connected by a linker that shows the residual secondary structure and fast local motion on the picosecond time scale [183,190]. NMR-based secondary chemical shift analysis and NOE data indicate that the linker adopts low populated extended structures analogous to polyproline II-like conformation [183]. Rotational correlation times (τ_c_) derived from NMR relaxation experiments for ASC^FL^ and the individual domains suggest that both domains reorient at different rates, but feel the drag from each other due to the presence of the linker [183].

### 4.1. Structural Details of ASC^PYD^ and Its Self-Association

ASC^PYD^ adopts the classic six-helical bundle motif typical of the DD-fold showing a long loop between helices α2 and α3, a unique feature commonly found in PYDs [167,189] (Figure 4B). The electrostatic surface of ASC^PYD^ is highly bipolar, with helices α1 and α4 containing mainly negatively charged residues, whereas helices α2, α3, and the connecting loop mostly accommodate positively charged residues [189]. Charge complementarity and the corresponding charge–charge interactions resulting from the bipolar distribution of the electrostatic surface are responsible for ASC^PYD^ self-associations. Two oppositely charged surfaces of ASC^PYD^ assemble back to back and self-associate with K_D_ = 40–100 μM using the dominant type I interaction mode, resulting in a buried surface area of 880 Å^2^. In the type I interaction for ASC^PYD^ self-association, helices α1 (E13), α4 (D51), the N-terminus of helix α5, and the α3-α4 loop (D48) of one surface interact with helices α2 (K21), α3 (R41), and the C-terminus of helix α5 of the opposite surface [112,167,189]. ASC^PYD^ displays a structural difference compared to other PYDs such as NALP1^PYD^ and NALP10^PYD^, as the latter shows considerable variation in the length of helices α1 and α6, and helix α3 is replaced by a disordered loop that may dictate their exclusive inflammatory functions [183].

Mutations of hydrophobic residues located in the PYD disrupt PYD-PYD filament formation, significantly increasing solubility at neutral pH while still retaining the monomeric folded conformation [190,191]. Similarly, NMR studies of ASC mutants in residues located in the type I and type III interfaces show complete or partially reduced ability of filament formation [167,190,192]. The L25A mutation in ASC^PYD^ has been commonly used in structural and biophysics studies to avoid oligomerization. NMR-based chemical shift analysis showed that the L25A mutation causes structural perturbations around residues K24 and L45 located at the α2-α3 binding interface. Structural perturbation around K24 destabilizes the α3-helix, hence diminishing ASC^PYD^ oligomerization by reducing the PYD binding ability. However, it is able to form dimers via the α1-α4 interface [112,190].

Cryo-EM studies revealed that ASC^PYD^ subunits pack densely in a helical tube-like, three-fold symmetry structure with 53° right-handed rotation and 14.0 Å axial rise consisting of six molecules per turn with inner and outer diameter of ~20 Å and ~90 Å, respectively [167,193]. NMR and cryo-EM based structural analysis of ASC^PYD^ and its comparison with other members of the Death Domain superfamily propose the involvement of all three interaction types in the stabilization of the PYD filament; i.e., intra-strand type I and inter-strand type II and III interactions [167,190]. These observations were further supported by mutagenesis experiments [182]. The type II interaction mode with a buried surface area of 524 Å^2^ involves contacts between helix α4 and the α4-α5 loop of one surface with the α5-α6 loop of the opposite surface. The type III interaction mode with a buried surface area of 360 Å^2^ involves contacts between helices α2 and α3 of one surface and the α1-α4 loop on another surface [167,182]. NMR and analytical centrifugation studies show that ASC^PYD^ polymer formation is favored in the presence of salt. Cryo-EM and solid-state NMR experiments conducted on mouse ASC^PYD^ with 71.8% sequence similarity to human ASC^PYD^ reveal very similar polymer structures [182,194].

### 4.2. Structural Details of ASC^CARD^ and Its Self-Association

**Figure 5:**
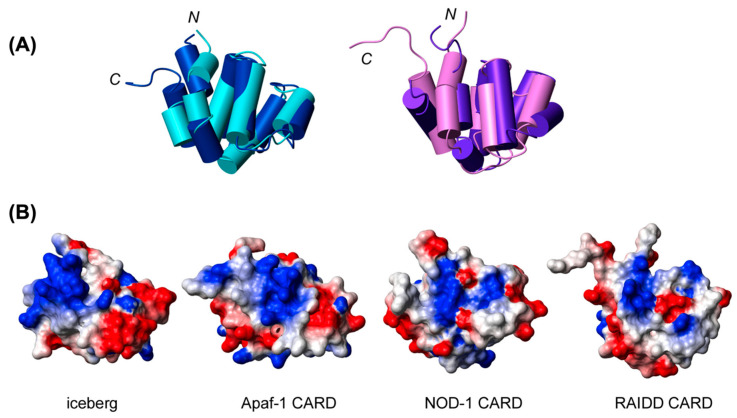
Structure comparison of caspase-activation and recruitment domains (CARDs) from different origins. (**A**) Superposition of Apaf-1-CARD (dark blue) [195] and iceberg (light blue) [199]. Superposition of RAIDD-CARD (pink) [200] and NOD-1-CARD (purple) [201]. Helix 1 (cylinder) in RAIDD-CARD, although not fragmented, is substantially bent and is shown as two cylinders; (**B**) electrostatic surface representation of CARDs in the same orientation as displayed in A. This figure is adapted from Figure S4 of [183].

CARDs adopt a conserved six-helix bundle fold and exclusively exhibit helix α1 divided into two small fragments, α1a and α1b, connected by a hinge [183,195,196]. Apaf-1 (Apoptotic protease activating factor), NOD1, ICEBERG, and RAIDD (RIP-associated ICH1/CED3-homologous protein with a death domain) are structurally homologous proteins, and structural comparison of their CARDs (Figure 5A) indicates differences in the length and orientation of the helices [183]. Among these, NOD1^CARD^ exhibits an extended long helix composed of helices α5 and α6 due to their close proximity [183]. Electrostatic surface analysis of these CARDs shows polarized distribution of basic and acidic surfaces, which dictates specific protein–protein interactions Figure 5B [197]. NMR experiments reported the absence of a fragmented helix α1, variability in length and orientation of the helices and evenly distributed charge in the electrostatic surface of ASC^CARD^ [183] (Figure 4B). ASC^CARD^ self-oligomerizes with a dissociation constant of 50 μM [112,198]. This NMR study indicates that residues located in the turn preceding helix α1 and helices α2, α3, α5, and α6 are involved in the self-association of ASC^CARD^, and generate three contact regions involving; (1) the N-terminus of helix α1 and C-terminus of helix α6; (2) the C-terminus of helix α5 and the N-terminus of helix α6; and (3) helices α2 and α3 [190]. Negatively stained TEM images illustrate that ASC^CARD^ assembles into two types/levels of filaments; ~3.4 ± 0.5 nm wide filaments that self-assemble into ~10 ± 0.5 nm wide bundles of filaments [190,198]. These NMR and TEM studies conclude that ASC^CARD^ plays a key role in the structure and stabilization of ASC filaments [190].

In addition, cryo-EM studies reveal that ASC^CARD^ can also polymerize into a helical tube-like filament with a diameter of ~8 nm and 3.6 subunits per turn, stabilized by type I, II, and III interactions [190,198,202,203]. The most predominant type I interaction involves charge–charge contacts between helix α2 (R119) in the surface of one protomer, and helices α1 and α4 (E130, D134 and R160) of the adjacent protomer. Hydrophobic residues such as W169 and Y187 participate in type II and III interactions. The type III interaction is also dominated by charge–charge interactions between R160 of helix α4, and D143 and E144 of helix α3 [202]. In the case of CARD polymerization, mutations involving residues that participate in type I (R119D, N128A/E130R, and D134K), type II (W169G, Y187A, Y187K), and type III interactions (D143K/E144K and R160E) completely abolish filament formation [202]. Analogously, mutations of E130, W131, and D134 by alanine impede the ability to oligomerize and thus ASC foci formation. These mutants form instead short and thin filaments as compared to WT ASC^CARD^. In addition, non-conservative mutations of negatively charged residues such as E130, D134, D191, and E193 by arginine completely impede filament formation [190].

### 4.3. ASC Filament Formation

TEM analysis of the dimensions of filaments and filament bundles formed by ASC^FL^ and the individual domains, PYD and CARD, indicate that both domains form an integral part of the ASC filament, thus elevating the role of ASC^CARD^ in filament formation, which has not been recognized in different studies of the truncated protein carrying only the PYD domain. In high-resolution TEM images, it is possible to discern the presence of stacked rings with an average diameter of 5 ± 0.6 nm, close to the dimensions of the experimentally-derived model of human ASC^FL^ dimer of 6 nm [198]. In addition, single-molecule FRET experiments also reported that both domains in ASC form fibrils in which the CARD folds back onto the PYD domain [204]. These results altogether show that the ASC dimer serves as a building block for ASC oligomerization, and both PYD and CARD domains are crucial for filament assembly. These latter studies proposed that speck formation has two levels of compaction; firstly, type I interactions mediates homophilic PYD-PYD and CARD-CARD binding, and secondly, type II and III interactions organize ASC into larger assemblies [182]. Computational and FRET studies suggest that ASC speck formation is not simply unspecific aggregation, but instead self-association follows an organized scaffold [182]. To confirm the involvement of both PYD and CARD domains in ASC speck formation, mutagenesis studies have been conducted. Single-point mutations in ASC (human) of residues important for the specific domain interactions such as E13A, E19A, K21A, K26A, R41A, D48A, D51A, L68A, L73A, in the PYD region, and M159A and R160A in the CARD, disrupt filament as well as ASC speck formation when present in ASC^FL^ [112,182,191,192,205,206]. Double mutations (K26A-R160A and L68A-R160A) generated in both CARD and PYD domains result in an inability to form filaments as well as ASC specks [182]. These data suggest that speck formation is due to the individual homophilic interactions mediated by the PYD and CARD. Altogether mutational, dynamics and structural studies of human ASC propose that speck formation is based upon two levels of compaction: One level involving a main type of homophilic interaction between PYD-PYD and CARD-CARD, and a second level organized by other interaction modes (e.g., type II and III interactions) [182].

These results match previous NMR studies on the 3D structure determination of ASC and its proposed model for polymerization indicating that the PYD-PYD and CARD-CARD domains are positioned in a confined space so that they do not cause steric interference with the binding interface of each other [183]. Dynamics resulting from a slightly structured linker lead to a back-to-back orientation of the two domains that increase the accessible space to facilitate the interaction of both PYD and CARD with the PYD of the sensor and the CARD of procaspase-1 [183,190].

### 4.4. Regulation of ASC

#### 4.4.1. Regulation of ASC Mediated by ASC2

Humans encode for 10–13 kD single domain PYD-only proteins (POPs) such as POP1/ASC2, POP2 and POP3, and CARD-only proteins (COPs) such as Pseudo-ICE/COP, ICEBERG, INCA. ASC2 shares 63% sequence identity with ASC^PYD^ [189,207,208,209], interferes with PYD-PYD interactions of inflammatory proteins, and has been shown to be crucial for modulating NF-κB and pro-caspase-1 regulation [208]. ASC2 binds to ASC^PYD^ with K_D_ = 4.08 ± 0.52 μM and serves as a negative regulator of ASC polymerization [209]. The L25A mutant of human ASC^PYD^ is capable of interacting with ASC2 (K_D_= 3.81 ± 0.8 μM) via the α1–α4 interface, which indicates that L25A mutation does not affect ASC^PYD^-ASC2 interaction at least in one of the possible interacting interfaces [209]. Site-directed mutagenesis of residues located in helices α2 and α3 of human ASC^PYD^ (K21, L25, K26, P40, and R41) disrupts ASC^PYD^ self-association without disturbing the hydrophobic pocket, thus indicating that ASC2 binding site on ASC^PYD^ is different from the site of self-association [191,209]. ASC2 displays a different orientation of helices α2 and α4 and has a disordered α3 helix. ASC2^PYD^ and ASC^PYD^ share similar 3D structures (RMSD = 1.5 Å) with comparable charge distributions across the surface [209]. NMR data indicate that the positively charged residues, K21 and R41, located on helices α2 and α3 of ASC2 interact via the type I interaction with the negatively charged residues D6, E13, D48, and D54 located on helices α1 and α4 of ASC^PYD^ [209].

#### 4.4.2. Post-Translational Modifications of ASC

Phosphorylation-dephosphorylation events are also important for ASC oligomerization and its activity. Tyrosine kinase-mediated phosphorylation of human ASC at Y60, Y137, and Y146 (Y144 in murine ASC) is necessary for inflammasome assembly and subsequent inflammatory response [210]. Similarly, the dephosphorylation of ASC tyrosine residues is also essential in the activation of the NLRP3 inflammasome. For example, it has been reported that the compound phenylarsine oxide (PAO), a tyrosine phosphatase (PTPase) inhibitor, suppresses ASC oligomerization and speck formation in LPS-primed human THP-1 cells by targeting the self-association nucleation step [210].

Differential ubiquitination of ASC by the K63 ubiquitin chain or linear ubiquitin plays an important role in ASC inflammasome activation. A ubiquitination enzyme complex LUBAC (linear ubiquitin chain assembly complex), which consists of HOIL-1, HOIP, and SHARPIN (Shank-associated RH domain-interacting protein) proteins, participates in linear ubiquitination of ASC via HOIL-1, HOIP E3 ligase activity [211,212] providing an activation signal. Another study reports that MAVS protein (mitochondrial antiviral signaling protein) recruits an E3 ligase, TRAF3 (TNF receptor-associated factor 3), that promotes ubiquitination of ASC at K174 position, which in turn increases ASC speck formation and secretion of IL-1β in response to viral infection [213]. In addition, it was found that a mitochondrial E3 ubiquitin ligase (Mul1 or MAPL or MULAN) abolishes inflammasome activation by K48-linked ubiquitination and subsequent proteasomal degradation of ASC [214].

## 5. Caspase-1

### 5.1. Structure and Activation of Caspase-1

**Figure 6:**
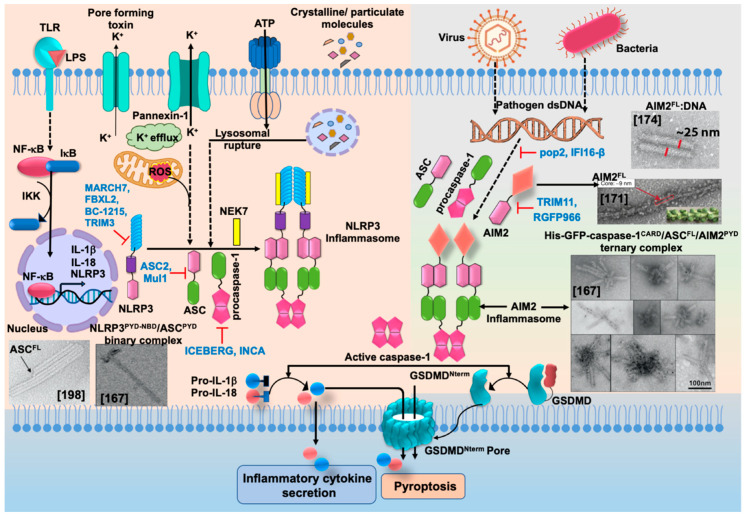
Schematic representation of NLRP3 and AIM2 inflammasomes activation and assembly. Pathogen-associated molecular patterns such as LPS, crystalline/particulate ligands, K^+^ efflux, and ROS trigger the activation of NLRP3. TLR initiates the activation and nuclear translocation of NF-κB, which increases the synthesis of NLRP3 and IL-1β and IL-18 cytokines. AIM2 detects viral and bacterial dsDNA in the cytosol. Assembly of NLRP3 or AIM2 with ASC and procaspase-1 leads to the proximity-induced autoproteolytic maturation of caspase-1, functionalization of IL-1β and IL-18, and pyroptosis cell death mediated by the N-terminal fragment of gasdermin D. Inhibitors of NLRP3, ASC, caspase-1, and AIM2 are shown blue. The reference sources corresponding to the negative-staining electron micrographs are shown in square brackets in each image: ASC^FL^ adapted from Figure 6 of [198], filamentous NLRP3^PYD-NBD^-ASC^PYD^ binary complex adapted from Figure 2 of [167]; filament of AIM2^FL^ from Figure 6 of [171], AIM2^FL^ filament with 600 bp dsDNA adapted from Figure 3 (Copyright National Academy of Science) of [174], and His-GFP-caspase-1^CARD^/ASC^FL^/AIM2^PYD^ ternary complex adapted from Figure 6 [167].

Caspase-1 (ICE; interleukin 1β-converting enzyme) is an inflammatory initiator that belongs to the aspartate-specific cysteine protease family [215,216,217]. It is expressed as a 404 amino acid-long inactive monomeric form called procaspase-1 zymogen, which is converted into a catalytic active form by autoproteolysis upon proximity-induced association mediated by the macromolecular organization of the inflammasome or ASC pyroptosome [218,219,220,221]. Procaspase-1 consists of one prodomain (or propeptide) CARD (1–119) that interacts with upstream adaptor proteins and a catalytic domain consisting of subunits p20 (120–297) and p10 (317–404) [167,222,223]. Caspase-1 activation involves proteolytic removal of the N-terminal CARD and 19 residues of the interdomain linker (298–316) connecting the p20 and p10 subunits [218,224]. Although the catalytic residues C285 and H237 reside in the p20 subunit, both subunits are essential for the activity [225]. X-ray crystallographic studies revealed that a tetramer of two p20/p10 heterodimers is considered as the functional form [223,226]. In contrast, recent cellular studies have shown that caspase-1^FL^ (p46) and transient species p33/p10 are dominant in the initial response to inflammasome assembly [219]. According to these studies, caspase-1^FL^ (p46) is recruited to the inflammasome via CARD-CARD interaction and generates an active p46 dimer, which then is self-processed and enables the cleavage of the linker connecting the p20 and p10 subunits to generate p30/p10 active species. Subsequently, the separation of the CARD domain linker (CDL) from p33/p10 releases the unstable p20/p10 tetramer (catalytic domain) from the ASC-caspase-1 complex, leading to the formation of the caspase-1 active form, therefore triggering the inflammatory response [219]. Activated caspase-1 facilitates the maturation of pro-IL-1β and pro-IL-18 into their bioactive forms IL-1β and IL-18, respectively [227,228] (Figure 6). Cytokine IL-1β induces the proliferation, activation, and differentiation of immune cells and facilitates phagocytosis, degranulation, and oxidative burst activity [229,230]. IL-18 is an inducer of IFN-γ and is involved in the activation and differentiation of various T-cell populations [231,232]. In addition to cytokine production, caspase-1 also cleaves gasdermin D (GSDMD) into two subunits of approximately similar size: N- and C-terminal halves. The GSDMD^Nterm^ creates pores in the plasma membrane that are involved in cytokine secretion and facilitates cell-death by pyroptosis [224,233,234,235,236,237] (Figure 6). It has been shown that before CDL cleavage, dimerized caspase-1 retains its catalytic activity towards pro-IL-1β, pro-IL-18, and pro-gasdermin D, but once the CDL is detached caspase-1 protease activity deteriorates [219].

When separated from the rest of the protein, caspase-1^CARD^ is able to polymerize into left-handed helical-tube macrostructures comprising four subunits per turn and with inner and outer diameters of ~10 Å and ~80 Å, respectively [231,238]. The formation of caspase-1^CARD^ filament also involves the interaction between the three interfaces [231]. Caspase-1^CARD^ filament shares helical symmetry with MAVS^CARD^ filament and Myddosome DD complex. Fluorescence polarization results suggest that caspase-1^CARD^ polymerization increases in the presence of ASC^CARD^ or ASC^FL^ [167]. Oligomerized ASC^CARDs^ nucleate procaspase-1 through CARD-CARD interaction thus serves as a platform for polymerization, autocleavage, and caspase-1 activation [167,239,240,241].

### 5.2. Negative Regulation of Caspase-1 Activation

CARD-only proteins (COPs) inhibit inflammasome assembly and cytokine activation [242,243,244]. These inhibitors include COP-1 (Pseudo-ICE/CARD16), INCA (CARD17), and ICEBERG (CARD18), and share high sequence identity with caspase-1 CARD, i.e., 92%, 81%, and 53%, respectively [199,231,244,245]. COP-1 and ICEBERG can self-associate and form filaments, whereas INCA harbors monomer conformation [231,246]. In vitro and in vivo experiments show that ICEBERG is involved in the negative feedback of caspase-1 activation, and therefore suppresses IL-1β secretion [199]. ICEBERG has the ability to inhibit the interaction between RIP2 (Receptor-interacting protein 2, also known as or RIPK2 or RICK) and caspase-1^CARD^, and also dissociates the preformed RIP2:caspase-1 complex [199].

It was proposed that the negatively charged surface of ICEBERG exhibits competitive binding for the positively charged surface of caspase-1^CARD^ with the upstream activator RIP2, which has a negatively charged patch [199]. Another study found that ICEBERG is not able to interact with RIP2, but COP1 can do so [231,246,247,248,249]. In vivo assays showed that both ICEBERG and COP1 hamper the binding of RIP2 to caspase-1 and reduce IL-1β expression by ~80% and 100%, respectively [246]. Two mutations, D27G and R45D, were created in caspase-1 in order to mimic the polypeptide sequence of INCA and ICEBERG [245]. These mutants were unable to activate NF-κB signaling due to loss of caspase-1 CARD-CARD interaction [245].

It has been reported as well that INCA inhibits caspase-1^CARD^ polymerization at nanomolar concentration even in the presence of ASC^CARD^. The mechanism proposed for this inhibition involves the capping of the growing caspase-1^CARD^ filament via CARD-CARD interaction, thus blocking the binding of upcoming caspase-1 molecules. This process would abrogate full polymerization of the caspase and subsequent autoactivation [231]. On the other hand, it was found that ICEBERG neither interacts with caspase-1^CARD^ nor inhibits NLRP3 inflammasome activation, caspase-1 oligomerization and its activity [231]. These findings suggest that cellular and/or environmental factors may influence ICEBERG-mediated inflammasome inhibition. Therefore, comprehensive studies are required to unravel the structural and functional mechanisms governing these inhibitory processes [231].

## 6. Formation of Inflammasome Assembly

### 6.1. NLRP3-ASC Interaction

Electron microscopy results reveal that the localization of NLRP3^PYD^ at one end of the ASC^PYD^ filament indicates the nucleation of ASC^PYD^ polymerization. However, the PYD-NBD (NLRP3ΔLRR or NLRP3^PYD-NBD^) fragment of NLRP3 is more efficient in triggering ASC^PYD^ polymerization than the monomeric NLRP3^PYD^, suggesting that oligomerization by NLRP3 is crucial for ASC^PYD^ polymerization. ASC^PYD^ filament formation monitored by fluorescence polarization (FP) in the presence of different molar ratios of NLRP3^PYD-NBD^ and ASC^PYD^ reveal that one molecule of NLRP3ΔLRR can stimulate the polymerization of up to 1600 ASC^PYD^ molecules [167]. NMR studies on the homotypic interaction between ASC^PYD^ and NLRP3^PYD^ reveal the involvement of two opposite surfaces: i.e., helices α1-α4 (R10, Y11, E13, D14, V18-L20, A47, D48, V50, D51, K84, and D88) and α5 helix (T4, G35, I37, F59, G61, E63, W66, A67, V70, W71, A74, E89, and K91). Further, based on NMR titration results and docking experiments, four different types of binding interfaces have been predicted for the ASC^PYD^-NLRP3^PYD^ interaction that include ASC α1-α4/NLRP3 α1-α4, ASC α1-α4/NLRP3 α5, ASC α2-α3/NLRP3 α1-α4, and ASC α2-α3/NLRP3 α5 [112]. ASC^PYD^ uses the same interfaces for self-association and binding to NLRP3^PYD^ [112]. The interaction between ASC^PYD^ and NLRP3^PYD^ could result in the formation of hexameric ring structures stabilized by E15, K23, E64, and D82 residues of NLRP3^PYD^ [112]. Similar ring architectures were also reported for other proteins assemblies such as the apoptosome and oligomeric forms of NLRP1 and NLRC4 with variable rotational symmetries [28,250,251]. Furthermore, based on NMR chemical shift perturbation results, amino acids L25, V30, and L45 were identified to mediate in ASC^PYD^-NLRP3^PYD^ binding, thus highlighting the importance of hydrophobic interactions [112].

### 6.2. Interaction of ASC with Procaspase-1 and Formation of the NLRP3 Inflammasome

ASC^CARD^ is an integral part of the ASC filament and participates in speck formation [183]. In addition, co-expression of ASC^CARD^ with caspase-1 can also form foci similar to those formed by ASC^FL^ [206]. Procaspase-1^CARD^ has one negatively and one positively charged surface oriented in opposite sides of the domain, which facilitates the type I interaction that is prominent in the recruitment of procaspase-1^CARD^ by ASC^CARD^. Mutational studies reported key residues responsible for the formation of foci: R10 (α1), D27 (α2), E41 and K42 (α3), R55 and D59 (α4) on caspase-1^CARD^, and R125, E130, D134, Y137, E144, R160, and D191 on ASC^CARD^ [206,245,252]. The mutation of caspase-1^CARD^ residues D27 and R55 completely interrupts ASC-caspase-1 signaling [206]. It is interesting to note that except for residues D143 and Y146, the rest of the mutants designed to perturb the type I interaction abolish foci formation. However, these ASC mutants can interact with caspase-1^CARD^ and show oligomeric assemblies, albeit lacking the ability to propagate active signaling platforms, which results in concomitant loss of IL-1β secretion [206]. Mutations of residues D143 and Y146 do not destabilize the ASC^CARD^ structure and allow foci formation, thus reflecting that a network of side-chain interactions might stabilize ASC^CARD^-ASC^CARD^ binding even in the presence of mutations in the interface [196,206,252]. These results point out the crucial role of ASC^CARD^ in foci formation and ASC-dependent inflammasome signaling [196,206,252].

The type III interaction (R45) is essential for caspase-1^CARD^ auto-oligomerization and recruitment of RIP2 [245,252]. D27 (type I interaction) mutant of caspase-1^CARD^ does not compromise auto-oligomerization; however, it fails to activate NF-κB signaling [206,245]. Similarly, caspase-1^CARD^ R45 mutant can interact with ASC without affecting proteolytic activation, but fails to trigger NF-κB signaling [245]. These findings suggest the importance of two oppositely charged surfaces and the synergistic effect of R45 and D27 on RIP2-mediated activation of NF-κB signaling [206,245,252].

Immunoprecipitation and EM studies of the NLRP3 inflammasome triggered by monosodium urate (MSU) crystals in THP-1 cells indicate the formation of filamentous structures that cluster into ball-of-yarn-like particles upon overnight incubation [167]. Similarly, expression of eGFP-ASC in COS-1 cells against anti-ASC primary antibodies reveal the formation of a densely packed gigantic perinuclear punctum (~1–2 μm) in each cell [167]. Analogously to the AIM2-^PYD^/ASC/Caspase-1^CARD^ assembly, a macro protein complex formed by NLRP3^PYD^/ASC^FL^/Caspase-1^CARD^ also assembles into initial star-shaped structures [68,167,253]. Molecular modeling based on NMR data and supported by in-cell immunoprecipitation and in vitro reconstitution suggest that ASC^CARD^ monomers could form 6–7 member ring structures via the type I interaction to which procaspase-1^CARDs^ could stack, amplifying the disk-like structure while leaving the ASC^CARDs^ accessible for interaction through type I, II, and III interactions [252]. The stacking of ASC^FL^ onto this ring creates an additional ring of PYDs below the CARDs. Overall, this arrangement would help PYD:PYD and CARD:CARD self-association and its interaction with the NLRP3 and AIM2 sensors, and procaspase-1 [252]. The systematic formation of NLRP3-inflammasome has been illustrated in Figure 6.

### 6.3. Interaction of AIM2 with ASC and Formation of the AIM2 Inflammasome

Upstream cytosolic dsDNA sensor AIM2 induces the polymerization of downstream ASC via homotypic PYD-PYD interactions, which further activate procaspase-1 [167,238]. Filaments formed by AIM2^PYD^ and ASC^PYD^ exhibit similar helical symmetry, overall dimensions, and subunit organization [167,171,174].

EM results along with modeling studies show that the AIM2^PYD^ filament nucleates the polymerization of the ASC^PYD^ filament [174], resulting in the localization of the latter at the end of former [167]. Thus, although ASC^PYD^ can self-polymerize, the presence of AIM2^PYD^ enhances this process. Fluorescence polymerization experiments suggest that both AIM2^PYD^ and the complex between AIM2^FL^ and dsDNA participate in ASC^PYD^ filament nucleation. Although it was found that the length of dsDNA regulates the self-association and propagation of AIM2 and ASC^PYD^ polymerization, both of them can accelerate their assembly irrespectively of the presence or absence of dsDNA [174]. It has been suggested that AIM2^PYD^-ASC^PYD^ assembly generates a three-stage continuous signal amplification in which AIM2^PYD^, ASC^PYD^, and AIM2^PYD^-ASC^PYD^ complex filaments are all persistent. In order to initiate AIM2^PYD^-ASC^PYD^ assembly, a critical concentration threshold is required. Both AIM2^PYD^ and AIM2^FL^ can induce ASC^PYD^ polymerization in a concentration-dependent manner and such ability is augmented by 4-fold in the presence of dsDNA [174]. FRET assays reveal that pre-assembled ASC^PYD^ polymers accelerate AIM2^PYD^ polymerization as well as the assembly of AIM2^FL^ on dsDNA, resulting in the regulation of the AIM2-ASC^PYD^ assembly by positive feedback loops. The AIM2^PYD/FL^-ASC^PYD^ complex filament is highly stable and once formed, biochemical data suggest that it cannot be disassembled [174].

### 6.4. AIM2 Ternary Complex: AIM2^PYD^:ASC^FL^:GFP-Casp1^CARD^

To reconstitute the ternary complex, His-MBP-AIM2^PYD^, His-MBP-ASC^FL^, and His-GFP-caspase-1^CARD^ were mixed in a ratio of 1:1:3 and incubated with TEV protease to remove the MBP tags [167]. After purification of the ternary complex, AIM2 and ASC were subjected to immunogold labeling, whereas caspase-1^CARD^ was labeled with Ni-NTA-nanogold conjugate. EM results reveal the formation of a star-shaped ternary complex in which AIM2^PYD^ could nucleate the short filaments of ASC^FL^ [167]. ASC subunits localize at the center of the complex-forming short filaments or rings, whereas the concomitant polymerization of AIM^PYD^ and ASC^PYD^ form long filaments. These results point to a critical role of ASC^CARD^ in the control of ASC supramolecular assemblies, suggesting distinctive structural features of the latter compared to ASC^PYD^ [198]. In the ternary complex, caspase-1^CARD^ is observed along the arms of the stars, possibly polymerizing via interaction with ASC^CARD^ [167]. The presence of the flexible linker connecting the PYD and CARD in ASC could facilitate the interaction with caspase-1^CARD^ [183]. The formation of the AIM2 and NLRP3 inflammasomes has been illustrated in Figure 6.

## 7. Concluding Remarks and Perspective

Inflammasomes provide host defense from pathogens by means of IL-1β and IL-18 maturation and secretion. The complex and high-order oligomeric nature of inflammasome components pose significant challenges for protein expression by recombinant methods and for successful purification. Moreover, size and shape heterogeneity deter detailed structural investigation. However, recent advancements have provided new insights into the structure, function, activation, and regulation of NLRP3 and AIM2 inflammasomes. Structural techniques, including NMR, X-ray crystallography, and cryo-EM, in association with biophysical, biochemical, and cell-based studies, have revealed high-resolution structures that help to understand the molecular mechanisms of ligand/receptor-driven conformational changes, the release of auto-inhibition, oligomerization, and complex assembly, nucleation-induced polymerization of ASC and caspase-1, and further downstream signaling. PTMs play important roles in the control of inflammasome activation. Hence, PTM dysfunction leads to autoimmune diseases resulting from chronic inflammasome activation.

In spite of recent progress in inflammasome research, several fundamental questions still remain unanswered. For instance, the ligand-induced activation mechanism of NLRP3 and its contribution in pyroptosis, which in turn can cause serious injury to vital organs, are still unclear [254]. Little is known about the exact mechanism of phosphorylation and ubiquitin-mediated controlled activation of inflammasome components. AIM2 can detect damaged or mislocalized self-DNA, which is released into the cytosol due to the loss of nuclear envelope integrity resulting from the perturbation of cellular homeostasis [255,256]. However, detailed information on how AIM2 regulates the detection of self as well as foreign DNA is lagging. Similarly, NLRP3 is also involved in self-DNA sensing, but the exact mechanism of self-DNA induced activation is not known [257,258]. In addition to NLRP3 activation, NEK7 also regulates microtubule dynamic and spindle assembly during the cell cycle [259]. How this protein limits NLRP3 activity without affecting AIM2 activation during cell cycle progression is unknown. Potential roles of AIM2 in inflammasome-independent processes, such as neuronal morphology, anxiety, and memory of mice will uncover new functions of AIM2 [260]. Although remarkable progress has been made on the structure of inflammasome components and mechanism of ASC speck formation, more structural details are required to elucidate the whole ASC speck assembly consisting of receptor, the ASC adaptor, and caspase-1. Likewise, super-resolution microscopy studies at the cellular level are required to uncover the formation of endogenous specks and involvement of accessory proteins that contribute to speck size and organized shape in intact cells. In-depth investigation of these fundamental questions will open up new doors for the development of novel therapeutics, and faster and efficient anti-inflammatory therapies for the treatment of associated autoimmune and autoinflammatory diseases. Finally, the determination of specific structural components of SARS-CoV-2 involved in NLRP3 activation and decoding the subsequent downstream pathway that leads to cell death, will aid in the development of potential therapeutics for the treatment of COVID-19 in near future.

## Figures and Tables

**Table 1:** Structural and functional details of canonical and non-canonical inflammasomes.

Inflamma-some	Expression Site	Activation Signal	Diseases	Structure
**Canonical Inflammasomes**				
NLRP1 (NALP1, CARD7, CLR17.1, DEFCAP, VAMASI)	Adaptive immune cells and tissues, non- hematopoietic tissues	A/B toxin of *Bacillus anthracis* [31,32]*, Toxoplasma gondii* infection [33], Muramyl dipeptide [28]	Vitiligo-associated multiple autoimmune disease [34], NLRP-1 associated autoinflammation with arthritis and dyskeratosis (NAIAD) [35], palmoplantar carcinoma and familial keratosis lichenoides chronica (FKLC) [36]	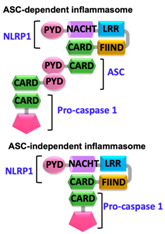
NLRP3 (Cryopyrin, NALP3, CIAS1, CLR1.1, PYPAF1)	Monocytes, neutrophils, dendritic cells, lymphocytes, osteoblasts, and epithelial cells	Pathogen-associated molecular patterns (PAMPs) (bacteria and virus) [37,38,39,40], danger-associated molecular patterns (DAMPs) such as Monosodium urate [41], alum [42], silica [43], asbestos [43], calcium/potassium efflux [44], extracellular ATP [39], reactive oxygen species (ROS) [45]	Cryopyrin-associated periodic fever syndrome (CAPS) [46], Muckle-Wells syndrome (MWS) [47], neonatal-onset multisystem inflammatory disease [48], Familial cold auto- inflammatory syndrome (FCAS) [49], Alzheimer’s disease [50] type II diabetes [51], cancer [52], chronic infantile neurological cutaneous and articular syndrome (CINCA, NOMID) [53]	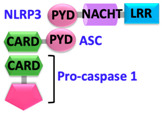
NLRC4 (IPAF, CARD12, CLR2.1)	Macrophage and intestinal epithelial cells	Bacteria [54,55], Cytosolic flagellin [56]	Syndrome of enterocolitis and autoinflammation associated with mutation NLRC4 (SCAN4) [57], Macrophage activation syndrome (MAS) [58]	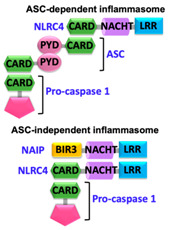
NLRP6	Cells of intestine and liver	Viral RNA [59], LTA of Gram-positive bacteria [60,61]	Colitis and colitis-induced tumorigenesis [62]	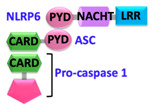
NLRP12 (RNO, PYPAF7, Monarch-1)	Neutrophils, eosinophils, monocytes, macrophages, and dendritic cells	Bacterial components [15,63,64]	Familial cold auto- inflammatory syndrome 2 (FCAS2) [65]	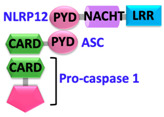
AIM2 (PYHIN4)	Cytosol of hematopoietic cells	Bacterial and viral dsDNA [66,67,68,69]	Psoriasis [70], abdominal aortic aneurysm [71], systemic lupus erythematosus [72], prostate and colonic cancer [73]	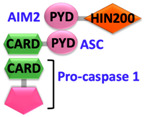
IFI16	Lymphocytes, monocytes, and epithelial cells	Viral and bacterial infections [74,75], Kaposi’s sarcoma-associated virus (KHSV) [76,77], HIV infections [78,79]	Systemic lupus erythematosus (SLE) [80]	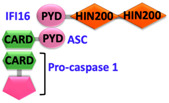
Pyrin (Marenostrin, TRIM20)	Neutrophils, eosinophils, monocytes, dendritic cells and synovial fibroblast	Bacterial infection [81], RhoA-GTPase inactivation [82,83]	Familial Mediterranean fever (FMF) [84], pyrin-associated autoinflammation with neutrophilic dermatosis (PAAND) [85]	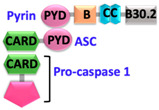
**Non-Canonical Inflammasomes**				
Human Caspase-4/5	Macrophages, epithelial cells, and monocytes	LPS of Gram-negative bacteria [4], Oxidized 1-palmitoyl-2-arachidonoyl-sn-glycero-3-phosphorylcholine (oxPAPC) [86]	Inflammatory bowel disease and colorectal cancer [87]	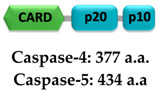
Mouse Caspase-11	Macrophages, epithelial cells, endothelial cells and neutrophils	LPS of Gram-negative bacteria [4], oxPAPC [5], Lipophosphoglycan (LPG) of *Leishmania* parasite [88], TIR-domain-containing adapter-inducing interferon β (TRIF) [89], secreted aspartyl proteinases [90]	Multiple sclerosis [91], Amyotrophic lateral sclerosis [92], Parkinson’s disease [93]. Inflammatory bowel diseases (IBDs) [94,95], Rheumatoid arthritis [86], Inflammatory respiratory diseases [96], Chronic obstructive pulmonary disease (COPD) [97]	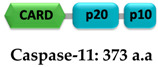

## Acronyms and Abbreviations

ADP	Adenosine diphosphate
AIM2	Absent in melanoma 2
ALRs	(AIM2)-like receptors
Apaf-1	Apoptotic protease activating factor
ASC	Apoptosis-associated speck-like protein containing a caspase-activation and recruitment domain (CARD)
Bcl	B-cell lymphoma
BIR	Baculovirus IAP (inhibitor of apoptosis protein) repeat
BMDCs	Bone marrow derived dendritic cells
BRCA1	Breast cancer type-1
BRCC3	BRCA1/BRCA2- containing complex, subunit 3
c-FLIP	FLICE-like inhibitory protein
CAPS	Cryopyrin-associated periodic syndrome
CARD	Caspase-activation and recruitment domain
CDL	CARD domain linker
CIAS1	cold induced autoinflammatory syndrome 1
CLR	C-type lectin receptor
COPs	CARD-only proteins
COS-1	CV-1 in Origin with SV40 genes
COVID-19	Coronavirus disease 2019
DAMPs	Danger-associated molecular patterns
DD	Death domain
DEDs	Death-effector domain
DEFCAP	Death Effector Filament-forming Ced-4-likeApoptosis Protein
DUBs	Deubiquitinating enzyme
EM	Electron microscopy
FADD	Fas-associated protein with death domain
FBXL2	F-box L
FBXO3	F-box O3
FLICE	Caspase-8/FADD-like IL-1β-converting enzyme
FP	Fluorescence polarization
FRET	Fluorescence resonance energy transfer
GFP	Green-fluorescent protein
GLMN	Glomulin
GSDMD	Gasdermin D
HD	helical domain
HDAC	Histone deacetylases
HIN	Hematopoietic, Interferon-inducible, Nuclear localization
IAPs	Inhibitor of apoptosis proteins
IC_50_	Inhibitory concentration
ICE	Interleukin 1β-converting enzyme
ICE	Interleukin-1beta converting enzyme
IFI16	Gamma-interferon-inducible protein
IFN	Interferon
IKK-γ	Inhibitor of nuclear factor kappa-B kinase subunit gamma
IL	Interleukin
INCA	Inhibitors of NFAT-calcineurin association
IPAF	Ice protease activating factor
IRF4	Interferon regulatory factor
ITC	Isothermal titration calorimetry
JNK	Jun N-terminal kinase
LPS	Lipopolysaccharide
LRRs	Leucine- rich repeat
LTA	Lipoteichoic acid
LUBAC	linear ubiquitin chain assembly complex
MALS	Multiangle light scattering
MALT1	Mucosa-associated lymphoid tissue lymphoma translocation protein 1
MAPK	Mitogen-activated protein kinase
MAPL	Mitochondria-associated protein ligase
MARCH7	Membrane associated ring finger (C3HC4) 7
MAVS	Mitochondrial antiviral-signaling protein
MBP	Maltose-binding protein
MLKL	Mixed lineage kinase domain like pseudokinase
MNDA	Myeloid cell nuclear differentiation antigen
MSU	Monosodium urate
Mul1	Mitochondrial E3 Ubiquitin Protein Ligase 1
NAIP	NLR family of apoptosis inhibitory protein
NALP	NACHT, LRR and PYD domains-containing proteins
NBD	Nucleotide binding domain
NEK7	NIMA-related kinase 7
NEMO	NF-kappa-B essential modulator
NF-κB	Nuclear factor kappa-light-chain-enhancer of activated B cells
NLRC	NLR family CARD domain-containing protein
NLRP	Nod-like receptor protein
NLRP3	NLR family pyrin domain containing 3
NLRs	NOD-like receptors
NMR	Nuclear magnetic resonance
NOD	Nucleotide-binding oligomerization domain
OB	Oligonucleotide/oligosaccharide binding)
PAMPs	Pathogen-associated molecular patterns
PAO	Phenylarsine oxide
PKA	protein kinase A
PKD	protein kinase D
POPs	PYD-only proteins
PP2A	protein phosphatase 2 A
PRRs	Pathogen recognition receptors
PTM	Post-translation modifications
PTPase	Protein tyrosine phosphatase
PTPN22	Protein tyrosine phosphatase nonreceptor type 22
PYCARD	PYD and CARD domain-containing
PYD	Pyrin domain
PYHIN	Pyrin + HIN
PYPAF1	PYRIN-containing APAF1-like protein 1
RAIDD	RIP-associated ICH1/CED3-homologous protein with a death domain
RICK	RIP-like interacting CLARP kinase
RIP2	Receptor-interacting protein 2
RMSD	Root-mean-square deviation
RNO	Regulated by nitric oxide
ROS	Reactive oxygen species
SARS-Cov-2	Severe acute respiratory syndrome coronavirus 2
SCF	Skp-Cullin-F box
SEC	Size-exclusion chromatography
SHARPIN	Shank-associated RH domain-interacting protein
STAT1	Signal transducer and activator of transcription
TEV	Tobacco etch virus.
T_H_2	T-helper cell type 2
TLR	Toll-like receptors
TMS	Target of Methylation-induced Silencing-1
TNF	Tumor necrosis factor
TPA	12-O-tetradecanoylphorbol-13-acetate
TRAF3	TNF receptor associated factor 3
TRIF	TIR-domain-containing adapter-inducing interferon-β
TRIM	Tripartite motif
WHD	winged helical domain
WHO	World health organization
